# Relapsing macrophage activating syndrome in a 15-year-old girl with Still's disease: a case report

**DOI:** 10.1186/1752-1947-3-138

**Published:** 2009-11-19

**Authors:** Meir Mizrahi, Eldad Ben-Chetrit

**Affiliations:** 1 Department of Internal Medicine A, Hadassah-Hebrew University Medical Center, POB 12000, Ein Kerem, Jerusalem, Israel

## Abstract

**Introduction:**

Macrophage activating syndrome is a severe, potentially life-threatening condition that may accompany Still's disease. It is characterized by fever, hepatosplenomegaly, lymphadenopathy, severe cytopenia, serious liver dysfunction, coagulopathy and neurologic involvement. The principal treatment for patients with this syndrome includes etoposide 150 mg/2 M twice a week for two weeks, dexamethasone 10 mg/2 M for two weeks and cyclosporine 3 mg/kg to 5 mg/kg for a longer period. Cases of relapse of macrophage activating syndrome are relatively rare.

**Case presentation:**

We describe the case of a 15-year-old Iraqi girl with Still's disease who developed macrophage activating syndrome with acute respiratory distress syndrome that required resuscitation and mechanical ventilation. Following intensive treatment, including high dose steroids and cyclosporine, the patient improved significantly. Two weeks after cyclosporine was discontinued, however, she was readmitted with an acute relapse of macrophage activating syndrome manifested by spiking fever, arthralgias, maculopapular rash and leukocytosis. This time the patient recovered following the reintroduction of treatment with cyclosporine and the addition of mycophenolate mofetil (Cellcept).

**Conclusion:**

We believe that cyclosporine is a cornerstone for the treatment of Still's disease. We recommend continuing this medication for several weeks following the patient's clinical recovery in order to prevent macrophage activating syndrome relapses.

## Introduction

Macrophage activating syndrome (MAS) is a severe, potentially life-threatening condition that is characterized by fever, hepatosplenomegaly, lymphadenopathy, severe cytopenia, severe liver dysfunction, coagulopathy and neurological involvement [1]. The principal treatment for patients with this syndrome includes etoposide 150 mg/2 M twice a week for two weeks, dexamethasone 10 mg/2 M for two weeks and cyclosporine 3 mg/kg to 5 mg/kg for a longer period of time. Relapsing cases of MAS are relatively rare. However, there are several case reports that describe a relapse happening after a short treatment or after the dose of cyclosporine was decreased rapidly [1].

Still's disease (SD) is a rheumatic disease of unknown etiology characterized by prolonged fever, arthralgias and/or arthritis and maculopapular rash. One of its grave complications is MAS, which can occur in up to 15% of reported cases [2].

We describe a rare case of a young patient with SD who presented with an acute relapsing pattern of MAS. Following intensive treatment, she recovered and has remained free of symptoms for the past 20 months.

## Case presentation

A 15-year-old Iraqi girl was admitted with a fever of 39°C which lasted for more than two weeks. A week prior to admission, she had complained of a sore throat, fever and maculopapular rash on her limbs, abdomen, palms and back. The patient was treated with doxycycline 150 mg twice a day, but due to her prolonged fever she was hospitalized. On admission, her physical examination was unremarkable. However, her blood count revealed leukocytosis of 23,000 (4000 to 10,000/mm^3^), with 90% neutrophils, hemoglobin of 12.6 Gr% (12 to 16 Gr% with MCV 75 fl (77 to 91 fl)), erythrocyte sedimentation rate (ESR) at 92 mm/1 hour, C-reactive protein (CRP) at 16 (0-1 mg%) and serum ferritin level at 2267 ng/ml (10 to 120 ng/ml).

The patient's electrolytes, kidney and liver functions and coagulation profile were all normal. Her antinuclear antibodies, rheumatoid factor, c-ANCA, p-ANCA, anticardiolipin antibodies, lupus anticoagulant, antiparietal cell antibodies, anti-smooth muscle antibodies, anti-histone antibodies and anti-mitochondrial antibodies all showed negative results. Serological tests for CMV, EBV, HSV, HAV, HBV, HCV, HIV, adenovirus, parainfluenza, Coxsackie, influenza, *Toxoplasma *and *Brucella *were all negative. Serology for parvovirus was positive for IgG antibodies as well as for *Coxiella burnetii *antibodies (Q-fever). A total body computed tomography (CT) scan was performed but did not show any pathology except for a 16-cm enlarged spleen. Bone marrow (BM) biopsy showed no evidence of lymphoma or any other type of malignancy.

Based on the patient's clinical and laboratory studies, a diagnosis of Still's disease was made. Three days after admission to the hospital, her liver enzymes became elevated. Two days later, the patient complained of shortness of breath with saturation of 85% at room air. Her chest X-rays were compatible with acute respiratory distress syndrome (ARDS). Her serum ferritin level rose to 10,648 ng/ml; her CRP rose to 27.7; and her ESR was at 102 mm/1 hour. A complete blood count showed anemia with hemoglobin values of 7.7 Gr%, a platelet count of 75,000 Gr% and white blood cell count of 18,000 (neutrophils 93%).

The patient's liver function tests showed the following results: ALT 101 U/l (6 to 53 U/l), AST 369 U/L (2 to 60 U/l), alkaline phosphatase 329 U/l (40 to 200 U/l), GGTP 1055 U/L (10 to 80 U/l), total bilirubin 39 micromol/l (0 to 17 micromol/l), and LDH 10,800 U/l (300 to 620 U/l). Her blood gases showed PO_2 _76 mmHg (85 to 90 mmHg), PCO_2 _31.8 mm/Hg (30 to 44 mmHg), HCO_3 _17.1 mmol/l (18 to 24 mmol/l) and pH 7.34 (7.38 to 7.42). Her coagulation profile disclosed a new alteration in the INR (international normalized rate) with values of 1.57 (1 to 1.4) and hypofibrinogenemia of 90 mg% (normal values at 140 to 400 mg%). Polymerase chain reaction (PCR) tests for CMV, EBV and HSV were performed on the patient and the results were all negative. A second BM biopsy, which was performed in order to rule out malignancy (possibly indicated by spiking fever, arthralgias, sore throat, leukocytosis and maculopapular rash), revealed impressive hemophagocytosis. The serum level of beta 2 microglobilin was higher than 12,000 mg/ml (normal value <2000), serum IL-2 receptor (IL-2R) was 1000 IU/ml (normal value <500 IU/ml) and serum tumor necrosis factor (TNF) was lower than 20 pg/ml. The patient was transferred to the intensive care unit and was intubated and ventilated with high positive end expiratory pressure (PEEP) levels.

A diagnosis of SD associated MAS was made and the patient was treated with high-dose intravenous methylprednisolone 1 g daily for three days, cyclosporine 5 mg/kg daily and supportive noradrenalin due to her low blood pressure (50/30 mmHg). With this treatment her fever gradually diminished after two days and laboratory values showed a slow improvement: a decline in leukocytosis to normal range 9.5 (4-0 10E9/l) with 75% neutrophils, a decline in serum ferritin levels to 900 ng/ml (10 to 120 ng/ml), ESR at 30 mm/1 hour and CRP at 2.9. Her serum fibrinogen and the coagulation profile became normal. Her platelet count normalized after nine days of treatment. Her repeated serum level of beta 2 microglobilin declined to 2600 mg/ml (normal value <2000). Her serum IL-2R was 560 IU/ml (normal value <500 IU/ml) and her serum TNF was lower than 20 pg/ml.

Due to the patient's clinical and laboratory improvement, the dose of cyclosporine was reduced to 3 mg/kg and that of solumedrol was reduced to 100 mg three times daily. After 10 days, the patient was successfully extubated and discharged with oral treatment. Two weeks later the patient was readmitted due to spiking fever, arthralgias and maculopapular rashes on her palms. A physical examination revealed jaundiced eyes and her laboratory tests showed serious liver dysfunction, with ALT 791 U/l, AST 611 U/l, alkaline phosphatase 404 U/l, GGTP 2105 U/l, LDH 1683 U/l, and total serum bilirubin at 177 micromol/l. An abdominal ultrasonography revealed that the patient had a normal size liver with a 16.5 cm enlarged spleen and normal pancreas and common bile duct. Her kidneys also appeared normal. Repeat blood tests showed leukopenia of 3200, which further declined to 1100, hemoglobin 11 gr%, platelets 217,000, ESR 92, CRP 4 and ferritin 2680 ng/ml. Her lipid profile disclosed hypercholesterolemia of 15.1 mmol/l (normal range 3 to 5 mmol/l), HDL 1.66 mmol/l (normal value > 0.91 mmol/l), LDL 11.11 mmol/l (normal range 0 to 3.4 mmol/l) and hypertriglyceridemia 5.0 mmol/l (normal range 0 to 2.3 mmol/l).

PCR tests for CMV, EBV and HSV were also repeated and gave negative results. The biomarkers test was also repeated and showed her serum level of beta 2 microglobulin to be higher than 9000 mg/ml (normal value <2000). Her serum IL-2R was 850 IU/ml (normal value <500 IU/ml) and serum TNF was lower than 20 pg/ml. A liver biopsy was then performed and showed a mild chronic inflammatory infiltration with no evidence of bile tract enlargement, foamy macrophage (hypercholesterolemia hypertriglyceridemia state), mild sinusoidal fibrosis and macrophage with intracellular iron pigmentation.

The official interpretation of the patient's condition was that of non-specific changes. An additional BM biopsy with aspiration was performed, again indicating severe hemophagocytosis. The patient was treated again with cyclosporine 5 mg/kg, solumedrol 1 g for three days and mycophenolate mofetil 500 mg three times daily. With this treatment the clinical conditions and the laboratory tests of the patient improved and she was discharged with the treatment of oral prednisone, cyclosporine and mycophenolate mofetil.

For the last 20 months the patient has remained completely asymptomatic while being treated with prednisone 5 mg per day (after a very slow tempering) and cyclosporine 75 mg per 2 days. Eight months after she recommenced this treatment, the prescription for mycophenolate mofetil was stopped.

## Discussion

Still's disease (SD) was described for the first time in children by George Still in 1896. It is characterized by a fever of approximately 39°C which continues for more than seven days, arthralgias or arthritis of two weeks duration or longer, and macular and/or maculopapular rash which is non-pruritic and salmon pink in color. Sore throat, lymph node swelling, hepatomegaly and/or splenomegaly with abnormal liver function studies, may also be present [3]. Typically, laboratory studies show an elevated ESR, which is accompanied by leukocytosis with the predominance of granulocytes. Normocytic normochromic anemia with hemoglobin values less than 10 g/dl and reactive thrombocytosis are seen in the majority of patients. Altered liver function studies are relatively common. However, liver biopsy findings are usually non-specific [4].

The serum ferritin level is high in patients with SD, with values of 2,500 to 10,000 or even higher in 70% of patients reported. These high levels most probably reflect an acute phase response, since hepatocytes responding to inflammatory cytokines increase ferritin synthesis [5]. Since ferritin levels are not that high in other rheumatologic diseases, it was suggested as a candidate for a serologic marker for the diagnosis and monitoring of the response to treatment of SD. However, other studies suggest that the portion of glycosylated ferritin (less than 20% of the total value of ferritin) is a more specific finding for the diagnosis of SD, in contrast to other rheumatologic diseases where the value is much higher [6]. Interleukin-6, TNF alpha, interferon gamma and interleukin 18 may be elevated during the active phase of the disease, but serology for antinuclear antibody and rheumatoid factor are negative [7].

The etiology of the disease is unknown and a variety of infectious triggers have been suggested including rubella, echovirus 7, mumps, cytomegalovirus, Epstein-Barr virus, adenovirus, parainfluenza, parvovirus, Coxsackie, influenza, herpes, and hepatitis B and C [8]. Possible bacterial etiology including *Yersinia enterocolitica *and mycoplasma pneumonia has also been raised [9]. Genetic factors such as HLA-B17, B18, B35 and DR-2 have been suggested as a predisposition for SD [10].

The diagnosis of SD is made using major and minor criteria. However, it requires the exclusion of infectious mononucleosis or parvovirus B19 infection, malignancy (particularly lymphoma), or other rheumatologic diseases such as polyarthritis nodosa and systemic lupus erythematosus [2].

Macrophage activating syndrome (MAS) is a severe, potentially life-threatening condition which may complicate chronic rheumatic diseases, especially systemic onset juvenile chronic arthritis. MAS is characterized by fever, hepatosplenomegaly, lymphadenopathy, severe cytopenia, serious liver dysfunction, coagulopathy and neurologic involvement. Early diagnosis of this condition is important because of the disease's aggressive clinical course.

Clinical and biological features of MAS closely resemble reactive hemophagocytic lymphohistocytosis (HLH) and the disease is in fact considered today as a subclass of HLH (secondary), which is induced by heterogeneous disorders including infections, malignant tumors and medications such as gold therapy, Aspirin and other non steroidal anti-inflammatory drugs [11]. The exact underlying mechanisms of MAS have not yet been clarified.

The diagnosis of MAS in a patient known to have a rheumatic disease must be suspected when the patient shows signs of systemic derangement, fever, hepatosplenomegaly, bleeding tendency, leukopenia, thrombocytopenia, increase in liver enzyme values and coagulations disturbance [12]. Hypofibrinogenemia is one of the most important clues for the diagnosis of MAS since patients usually have high fibrinogen levels due to their underlying inflammatory disease. An increased fibrinolysis is most probably due to uncontrolled activation of the macrophages and the production of plasminogen. A decrease in factor II and factor VII + X values is also observed in some reported cases [13]. A bone marrow smear may show macrophage hemophagocytosis. Another biological indicator of MAS is an increased level of serum triglyceride that can be related to the patient's extensive production of cytokines, such as tumor necrosis factor alpha, which reduce the lipoprotein lipase activity. Interleukin-1 and interferon gamma are also overproduced in a patient with MAS [1].

In 1994, the Histocyte Society conducted the first international treatment study for patients with HLH and they recommended treatment with etoposide 150 mg/2 M twice a week for two weeks, dexamethasone 10 mg/2 M also for two weeks, cyclosporine 3 mg/kg to 5 mg/kg for a long period and metotrexate in cases where the patient's nervous system is already affected. In resistant cases, several modes of therapy were described including high dose steroids, cyclosporine, antihuman thymocyte globulin (ATG), intravenous immune globulin (IVIG), plasma exchange and allogeneic bone marrow transplantation [14]. Recently, IL-1 beta receptor antagonist (Anakinra) was used with success in severe cases of HLH [15].

## Conclusion

This case is remarkable for its special pattern of an acute relapsing MAS. The exact mechanism underlying the relapsing pattern of the disease is not well understood. However, our patient fully recovered following long-term treatment with steroids, mycophenolate mofetil and cyclosporine. We cannot evaluate the exact contribution of each medication to the patient's positive response. Nevertheless, we believe that the prolonged use of cyclosporine treatment played a major role in her recovery.

We recommend continuing treatment with cyclosporine for several weeks following the patient's clinical and laboratory recovery in order to prevent a MAS relapse. Treatment with IL-1 beta receptor antagonists should also be considered in future cases.

## Abbreviations

ALT: alanine transaminase; ARDS: acute respiratory distress syndrome; AST: aspartate aminotransferase; ATG: antihuman thymocyte globulin; BM: bone marrow; C-ANCA: granular anti-neutrophil cytoplasmic antibodies; CMV: cytomegalovirus; CRP: c-reactive protein; CT: computed tomography; EBV: Epstein-Barr virus; ESR: erythrocytes sedimentation rate; GGTP: gamma-glutamyl transferase; HAV: hepatic A virus; HBV: hepatic B virus; HCV: hepatic C virus; HDL: high-density lipoproteins; HIV: human immunodeficiency virus; IVIG: intravenous immune globulin; LDL: low-density lipoproteins; MAS: macrophage activating syndrome; P-ANCA: perinuclear anti-neutrophil cytoplasmic antibodies; PCR: polymerase chain reaction; PEEP: positive end expiratory pressure; SD: Still's disease; TNF: tumor necrosis factor; WBC: white blood cells.

## Consent

Written informed consent was obtained from the patient's parents for publication of this case report and any accompanying images. A copy of the written consent is available for review by the Editor-in-Chief of this journal.

## Competing interests

The authors declare that they have no competing interests.

## Authors' contributions

MM reviewed the patient's medical records and imaging findings, drafted the manuscript and coordinated the submission of this manuscript. EBC critically reviewed the manuscript and took part in treating the patient.
